# Heightened clinical utility of smartphone versus body-worn inertial system for shoulder function B-B score

**DOI:** 10.1371/journal.pone.0174365

**Published:** 2017-03-20

**Authors:** Claude Pichonnaz, Kamiar Aminian, Céline Ancey, Hervé Jaccard, Estelle Lécureux, Cyntia Duc, Alain Farron, Brigitte M. Jolles, Nigel Gleeson

**Affiliations:** 1 Physiotherapy Department, Haute Ecole de Santé Vaud (HESAV)//HES-SO, University of Applied Sciences Western Switzerland, Lausanne, Switzerland; 2 Service of Orthopaedics and Traumatology, Department of Musculoskeletal Medicine, University Hospital of Lausanne, Lausanne, Switzerland., CHUV-UNIL, Lausanne, Switzerland; 3 Laboratory of Movement Analysis and Measurement, Ecole Polytechnique Fédérale de Lausanne (EPFL), Lausanne, Switzerland; 4 Direction médicale, CHUV-UNIL, Lausanne, Switzerland; 5 School of Health Sciences, Queen Margaret University, Edinburgh, Scotland; Universite de Nantes, FRANCE

## Abstract

**Background:**

The B-B Score is a straightforward kinematic shoulder function score including only two movements (hand to the Back + lift hand as to change a Bulb) that demonstrated sound measurement properties for patients for various shoulder pathologies. However, the B-B Score results using a smartphone or a reference system have not yet been compared. Provided that the measurement properties are comparable, the use of a smartphone would offer substantial practical advantages. This study investigated the concurrent validity of a smartphone and a reference inertial system for the measurement of the kinematic shoulder function B-B Score.

**Methods:**

Sixty-five patients with shoulder conditions (with rotator cuff conditions, adhesive capsulitis and proximal humerus fracture) and 20 healthy participants were evaluated using a smartphone and a reference inertial system. Measurements were performed twice, alternating between two evaluators. The B-B Score differences between groups, differences between devices, relationship between devices, intra- and inter-evaluator reproducibility were analysed.

**Results:**

The smartphone mean scores (SD) were 94.1 (11.1) for controls and 54.1 (18.3) for patients (P < 0.01). The difference between devices was non-significant for the control (P = 0.16) and the patient group (P = 0.81). The analysis of the relationship between devices showed 0.97 ICC, −0.6 bias and −13.2 to 12.0 limits of agreement (LOA). The smartphone intra-evaluator ICC was 0.92, the bias 1.5 and the LOA −17.4 to 20.3. The smartphone inter-evaluator ICC was 0.92, the bias 1.5 and the LOA −16.9 to 20.0.

**Conclusions:**

The B-B Score results measured with a smartphone were comparable to those of an inertial system. While single measurements diverged in some cases, the intra- and inter-evaluator reproducibility was excellent and was equivalent between devices. The B-B score measured with a smartphone is straightforward and as efficient as a reference inertial system measurement.

## 1. Introduction

### 1.1. Current methods for shoulder function evaluation in clinical settings

The shoulder is the second most frequently affected body site [1]. The quality of tools for the evaluation of shoulder function is of primary interest to adequately address the problems of this large population and therefore limit the impact of shoulder pathologies on patients and society. Shoulder function is usually evaluated using questionnaires. Dozens of evaluation tools exist but most have not undergone a full validation process [2, 3]. Thus the measurement of the shoulder functional outcome remains a controversial issue.

Several reviews of literature have concluded that no single questionnaire of shoulder function offered superiority regarding measurement properties [3–5], while one concluded that the DASH (Disabilities of the Arm, Shoulder and Hand) score compared favourably to other questionnaires [6]. As a consequence, a large variety of outcome measurements tools have been used, hindering the development of scientific evidence about the treatment of shoulder conditions [2].

Clinical questionnaires have the advantages of handiness and low cost. Conversely, they present intrinsic limitations related to language and cultural issues, respondents’ interpretations and content validity [7, 8]. The validation of questionnaires’s translations into various languages is a time-consuming and cumbersome process. Moreover, the delineation between objective and subjective evaluation is not always clearly defined in questionnaire-based assesssment, with both approaches producing different results [9, 10].

### 1.2. Computerized shoulder function evaluation

Laboratory-based movement analysis overcomes these limitations and displays high accuracy and precision. It has thus been largely used in research studies aiming at the characterization and evaluation of shoulder motion. Most motion analysis studies have addressed the development of innovative measurement' methods mainly and have investigated differences between healthy and pathological participants’ groups. However, none of them had proposed a shoulder function score that could be possibly used to monitor patient clinical evolution, to the best of our knowledge.

Although 3D laboratory motion analysis systems have assumed a growing importance in research, it’s their application in clinical settings that has remained likely to be limited by complexity and cost. So, embedded systems, like inertial measurement units (IMU) have also been developed for shoulder evaluation, as their portability and practicality facilitates the procedures for measurement.

Measurements using embedded systems may provide a well-balanced compromise between practicality and reliability. They may thus constitute a valuable alternative to questionnaires or laboratory-based evaluation. The embedded systems’ results are highly correlated to laboratory measurements and display adequate accuracy for clinical evaluation. Also, their use is not restricted to laboratory settings and the measurement completion is easier [11]. Body-worn sensors have been applied with promising results, to measure arm and shoulder movement in various conditions [12–20].

Despite the simplification of the measurement procedures provided by body-worn sensors their use for shoulder function evaluation has remained limited in clinical settings. Several barriers still hinder the wide-spread use of such devices among health professionals. The requirements for the routine application in clinical practice are very demanding as, in addition to measurement properties, time, practicability, user-friendliness and cost are of concern.

Using a smartphone for evaluation purposes might contribute to meeting these requirements and facilitating the regular use of computerized movement analysis in current practice. Like embedded measurement systems, most smartphones are now fitted with built-in accelerometers and gyroscopes. Using a dedicated application, they can thus be used for movement analysis.

### 1.3. Present smartphone applications for shoulder evaluation

Numerous smartphone applications have been developed for patient evaluation, patient education or to assist health care professionals in their practice. The applications addressing the assessment of shoulder range of motion (ROM) generally demonstrated adequate measurement properties [21–23]. However, ROM is only one component of shoulder function and no smartphone-based assessment score for shoulder function has been validated to our knowledge. The validation of smartphone-based outcomes would be of interest because of the high prevalence of shoulder conditions and of the existing controversy about shoulder function questionnaires.

Smartphone-based evaluation in clinical conditions is valuable only provided that the measurement properties have previously been validated. This is mandatory as important decisions are taken based on clinical outcome. The smartphone results might possibly differ from inertial-based systems as the sensors’ features have not been specifically designed for scientific measurement. An extensive validation process is thus needed before clinical implementation.

### 1.4. Inception of a smartphone application for shoulder function

Coley developed a shoulder function scoring system using inertial sensors. He proposed a relatively simple shoulder function score based on three dimensional measurements of a power-related metric using accelerometers and gyroscopes (P score) [11]. The procedure relied on a sequence of seven functional movements based on the Simple Shoulder Test functional score [24]. This approach demonstrated clinical relevance following rotator cuff and arthroplasty surgery. It clearly discriminated healthy from pathological subjects, was correlated to clinical scores and displayed good responsiveness [11]. However, the full test procedure required around 20 minutes, which precluded routine application in clinical settings.

Körver et al. [25, 26] proposed a kinematic score based on angular rate (AR Score). This score required less than 5 minutes to perform as it included only “arm to the back” and “arm behind the head” movements. It demonstrated high intra- and inter-evaluator reproducibility, with intraclass coefficient of correlation (ICC) of 0.95 and 0.91, respectively. The diagnostic sensitivity was 98% and the specificity 81%. However, the criterion-based validity for shoulder function evaluation was limited, as correlations with the DASH and SST (simple shoulder test) clinical scores were weak [24, 27].

The latter weakness was not found for the B-B Score, a simplified version of P Score including two movements only (hand to the Back & hand upwards as if to change a Bulb) [28]. This score was developed based on principal component analysis and multiple regression of the P Score original data. The B-B Score results showed no significant difference with the P score during the first year after shoulder surgery and both scores were highly related (R^2^ >.97). The diagnostic sensitivity was 97% and the specificity 94% for patients following rotator cuff surgery or shoulder arthroplasty. The correlations with current clinical questionnaires ranged from 0.51 to 0.77, indicating that the B-B Score had good criterion-based validity for shoulder function evaluation. Thus, the simplified model is comparable to the P Score but presents practical advantages that facilitate the evaluation of shoulder function in clinical practice.

Pichonnaz et al. [29] investigated the measurement properties of a smartphone-based version of the B-B Score in various shoulder pathologies. Diagnostic power, responsiveness and concurrent validity with shoulder function questionnaires were insufficient for shoulder instability, but were appropriate for patients conservatively treated for rotator cuff conditions or capsulitis, and patients surgically or conservatively treated for proximal humerus fracture, when compared to accepted clinimetric standards.

Despite these promising results, it remains presently unknown if the measurement obtained using a smartphone are comparable those obtained using a reference human movement analysis system and display equivalent reproducibility. If so, the use of a smartphone for the B-B Score measurement might offer a cost-effective and straightforward clinical outcome measurement.

### 1.5. Study aim and hypotheses

The aims of this study were to investigate the validity and reproducibility of a smartphone-assessed kinematic shoulder function B-B Score, and to compare the performance of the smartphone to a reference inertial system.

Thus, the study hypothesis is that the B-B Score meets the requirements of a valid shoulder function score. This implies that the differences between the control and the pathological group but not the difference between devices should be significant, the ICCs ≥ 0.80 for inter-device, intra-evaluator and inter-evaluator reproducibility, the limits of agreement (LOA) between devices ≤ 10% and the bias ≤ 5% [30, 31]. The B-B Score results should also be coherent with those of shoulder function questionnaires.

## 2. Materials and methods

### 2.1. Study sample

A prospective cohort study was conducted between August 2011 and May 2014 at the Department of Traumatology and Orthopaedic Surgery of the University Hospital of Lausanne. Ethical approval was granted by the Human Research Ethics Committee of the Canton of Vaud (CER-VD), protocol number 205/10. Patients gave their signed informed consent for participation in the study. The study was registered under ClinicalTrials.gov Identifier: NCT01431417. Three healthy participants where inadvertently measured within the two weeks preceding the registration date. The measurement protocol was strictly identical for all participants and was in line with study declaration.

The included patients were adults > 18 year old. They presented with one of the following shoulder conditions, as recorded during their first medical consultation at the specialized shoulder consultation unit of the hospital: rotator cuff condition, adhesive capsulitis, proximal humerus fracture i.e. the pathologies for which the B-B score measurement properties were known as appropriate [29]. With the exception of patients with fracture, patients who gave their consent underwent the measurement session within two weeks following medical consultation. Measurements were performed 6 weeks post stabilisation for patients with humerus fracture, provided that the radiological control showed normal consolidation.

For the rotator cuff condition or capsulitis, patients were selected who required only conservative treatment. As the B-B Score had previously been validated after rotator cuff and arthroplasty surgery [28], it was of interest to explore its validity in different populations. Surgical and conservative fracture treatment were included in the same group as the evolution and functional prognosis is similar in both populations [32].

A group of participants younger than 35 years-old without history of shoulder condition/pain, was also included to evaluate the performance in a healthy population and the stability of the score. These participants were selected purposefully to be younger than the patients to avoid bias related to the high prevalence of asymptomatic rotator cuff tear above 40 years old [33].

The sample size calculation was based on the data of a pilot study that included 7 controls and 16 patients. The calculation was made so that, with a significance level at P< 0.05, the power of 0.80 was reached when the minimal standards for acceptable properties of the score were met. Fourty-six patients were required considering a lowest acceptable ICC of 0.80, corresponding to a substantial correlation, and an expected ICC of 0.90 for two measurements [31, 34]. Nine patients were required to get the expected power for the difference between the patients and the control group [35, 36]. A considerably larger sample was enrolled to get precise estimations of results and to allow subsequent subgroup analysis in further investigations.

Exclusion criteria were bilateral shoulder conditions, any concomitant pain or condition involving the upper limb or cervical spine, medical contraindication to execute movements required for score completion, tumour, neurological condition interfering with the test and an insufficient local language level to give truly informed consent or to understand questionnaires.

### 2.2. B-B Score calculation

The B-B Score was calculated according to the method described in Pichonnaz et al. and Coley at al. [11, 28]. A power-related parameter was extracted from the recorded signals: the range of acceleration was multiplied by the range of angular velocity, with a measurement unit of [(deg/s) × (m/s^2^)], for each movement. This parameter was calculated for each axis and for each movement of the B-B Score (“hand to the Back” movement and “lift hand as to change a Bulb” movement) and added, separately for each side and for each movement. The ratio of the performance of the affected side relative to the healthy side (or the dominant side relative to the non-dominant side for healthy participants), expressed in percentage, was then calculated for each of the two movements. The values of the movements were then weighted using the equation: B-B Score = 16.71 + 0.32 x hand to the Back. + 0.45 x lift hand.

One hundred percent represents a perfect balance in capability between sides and the score decreases in accordance with the severity of functional loss. For example, while a typical healthy person performs near to 100%, the average patient might reach 46% before surgery, 67% at 3 months and 71% at 6 months after surgery.

### 2.3 Experimental system: Smartphone

**Fig 1:**
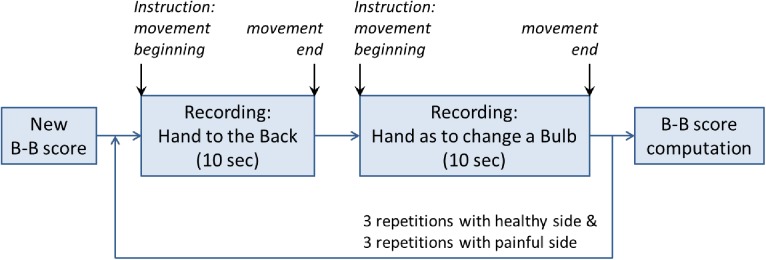
Schema of the application steps for the recording of a B-B score. From: Pichonnaz C, Duc C, Gleeson N, Ancey C, Jaccard H, Lecureux E, et al. Measurement Properties of the Smartphone-Based B-B Score in Current Shoulder Pathologies. Sensors (Basel). 2015;15(10):26801-17.

A smartphone (iPod^®^, Apple, Cupertino, USA) was chosen as the support device for the development of the application. It was fitted with 3D built-in sensors (Accelerometers: ± 2 g precision: ± 0.02 g; Gyroscopes: ± 500 deg./s precision: ± 0.2 deg./s; Sampling frequency: 100 Hz) [37]. An application, called iShould (instrumented shoulder test) was programmed in Objective-C [38, 39]. This application enabled the acquisition of the acceleration and angular velocity signals during the movements of the B-B Score and the computation of the B-B Score value, as described in the Fig 1. Once the application was launched, the smartphone provided instructions to the user, through the smartphone loudspeaker, when to perform a score movement. For each score movement, the application recorded the acceleration and angular velocity signals for a predefined period of 10 sec. The movements were first performed with the healthy side and then repeated with the painful side. At the end of the test, the B-B Score was directly calculated, displayed on the smartphone screen and then stored on the smartphone. The application enabled exporting of all saved data to a computer for its direct comparison with the data from the inertial sensors of the reference system.

### 2.4 Reference system

The reference system for body-worn movement analysis was composed of 2 inertial sensors and a datalogger system (Physilog®, Gait Up, Lausanne Switzerland).

Each inertial sensor included three dimensional accelerometers and gyroscopes (Accelerometers: Analog device, ADXL 210, ±5 g, precision: ± 0.2% of Full Scale; Gyroscopes: Analog device, ADXRS 250, ±400 deg/s, precision: ± 0.1% of Full Scale). The device resolution was 16 bits and the sampling frequency was 200 Hz.

An inertial measurement system was used as a reference in this study because the B-B Score has been previously developed based on this approach, and because inertial sensors provide direct measurements of angular velocities and accelerations used in the score calculation. Initial study try-outs showed that the influence of measurement errors (offset, sensitivity or drift) was negligible in the study context.

### 2.5. Measurement procedure

**Fig 2:**
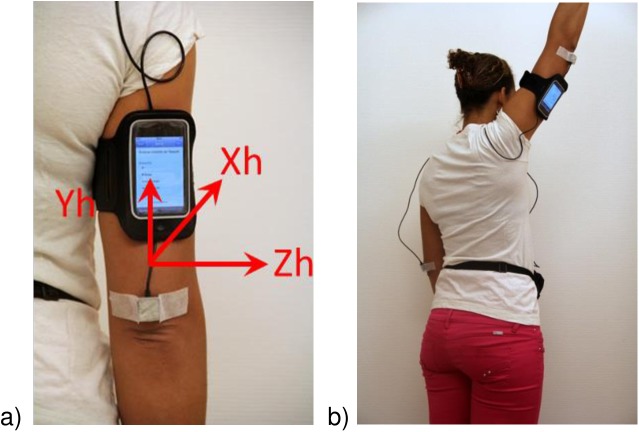
Inertial sensors and smartphone placement and axes. (a) The inertial sensor module (Physilog® reference system) attached to the arm with medical tape and connected by cable to the datalogger carried on wait. The smartphone is attached to the arm with the armband. (b) Test completion of “hand to the ceiling”.

The inertial sensors of the reference system were placed on each humerus, 3 cm above the midpoint of the line connecting the lateral epicondyle (EL) and medial epicondyle (EM). The sensor’s axes were aligned to the anatomical frame of the humerus following the ISB recommendations [40, 41]: Yh on the line connecting the gleno-humeral (GH) joint and the midpoint of EL and EM, pointing to GH; Xh on the line perpendicular to the plane formed by EL, EM and GH, pointing forward; Zh on the line perpendicular to Xh and Yh, pointing to the right (Fig 2). The smartphone was also attached to the back of the arm with an armband. The lower edge of the smartphone was set 3 cm above the upper edge of the inertial sensors’ module [29]. Similar to previous work angular velocities and accelerations in the sensor frame have been used to calculate the B-B Score [11, 28].

After setting-up of the systems, the participants watched a video-recorded demonstration of the execution of the B-B Score. They were instructed to do the movements in the pain free ROM, at their self-selected speed and in their natural way. The starting position was the arm alongside the body, in a relaxed position. Movements were executed in a standing position following the smartphone-recorded instructions. The patients undertook first 3 repetitions of the two B-B Score movements on the healthy side (put hand to the back + hand to the ceiling as to change a bulb) and then repeated the task on the pathological side. The controls executed the same procedure beginning on the dominant side.

The measurement procedure was repeated twice alternating between two evaluators. All evaluators were experienced physiotherapists engaged in the project, who had previously been trained to the score completion. The first evaluator was randomly assigned. All measurement systems were detached for inter-evaluator administration of assessments to account for the variability induced by possible inconsistent sensors’ placement in clinics. The score was calculated based on the mean of the 3 replications because the pilot study showed that the variability was not significantly different with a higher number of repetitions.

Clinical questionnaires were also completed. Three currently used shoulder function questionnaires [Quick Disabilities of the Arm and Shoulder score (QuickDASH), Simple shoulder test (SST), Constant score and Constant relative score (based on an age- and sex-matched normal populations)], the EuroQol generic quality of life questionnaire [EQ-5D] and the pain visual analog scale (VAS) [24, 42–44]. The Constant Score was undertaken according to the modified guidelines of Constant [45]. The shoulder function questionnaires were selected because they represent current standards [3, 4, 46, 47]. They allowed the evaluation of the concurrent validity for the B-B Score but not of its validity against a ‘gold standard’, due to the controversy surrounding shoulder function evaluation.

### 2.6. Analysis

Descriptive statistics including mean, standard deviation (SD) and boxplots were performed for patients’ characteristics and outcomes of both groups. The difference between the B-B Scores measured by each device was evaluated using the Wilcoxon rank-sum test. The relationship between the B-B Scores of each device, and the intra- and inter-evaluator reproducibility were evaluated using the ICC, measurement error (ME: standard error of the mean difference), standard error of measurement [SEM: $\left(pooled\;SD\times \sqrt{1-ICC\;agreement}\right)$] and Bland and Altman LOA analysis. Intra-evaluator reproducibility was calculated comparing the 1^st^ with the 2^nd^ score obtained by the same evaluator, for the two evaluators. Inter-evaluator reproducibility was calculated comparing the score obtained by one evaluator with the score by the other evaluator, for the 1^st^ and 2^nd^ evaluator’s measurement. The Shapiro–Wilk test and Komolgorov-Smirnov tests were used for the normal distribution analysis. The discriminative power was evaluated by the significance level for the differences between groups (Mann-Whitney) and between stages (Wilcoxon).

## 3. Results

### 3.1. Study sample

Twenty healthy participants and 65 patients (20 with rotator cuff condition, 23 with fractures, 22 with capsulitis) were included.

The population characteristics and the significance of the differences between groups are described in Table 1.

**Table 1:** Participants’ characteristics.

	**Patient (n = 65)**	**Control (n = 20)**
**Age mean (SD), years**	58.5 (14.2)**	28.2 (6.2)
**Sex (% women)**	63	50
**Weight mean (SD), kg**	75.2 (15.8)	74.7 (17.4)
**Body mass index mean (SD), kg/m^2^**	26.6 (5.8)	24.2 (3.9)
**Size mean (SD), m.**	1.68 (0.10)	1.75 (0.10)
**Hand dominance (% right-handed)**	92	90
**Affected side (% dominant side)**	43	-

** Significant difference between groups with p-value < 0.01.

### 3.2. Score outcome

**Fig 3:**
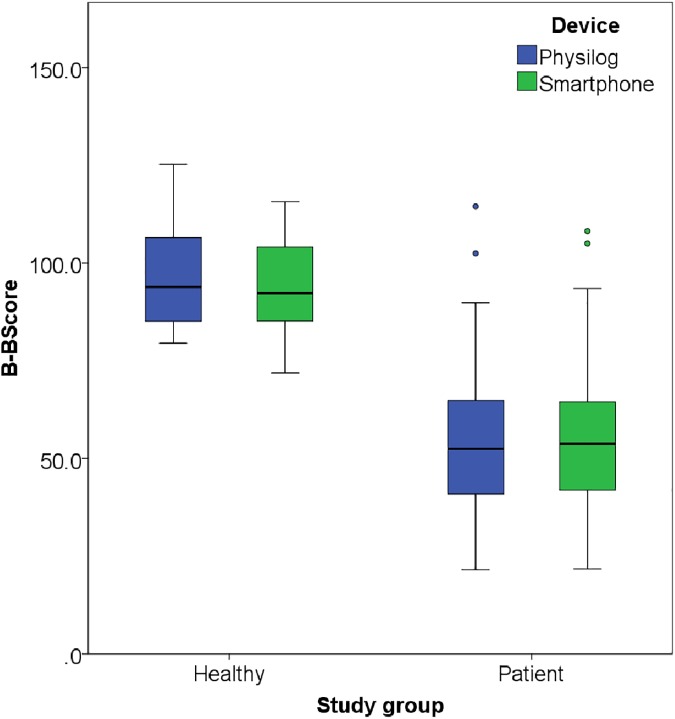
B-B Score outcome in both groups using the reference system (Physilog^®^) and the smartphone.

The outcomes of the control group and the patient group, for the smartphone and the reference system (Physilog^®^), respectively, are presented in Table 2 and in Fig 3.

**Table 2:** Mean and standard deviation of B-B Score using the smartphone and the reference system. Unit of scores are % representing the performance of the pathological side compared to the healthy side.

**Mean (SD), %**	**Reference system**	**Smartphone**
**Min;max**		
**Control**	97.0 (13.8)	94.1 (11.1)
	79.5 ; 125.2	71.9 ; 115.7
**Patient**	54.0 (19.0)	54.1 (18.3)
	21.5; 114.5	21.7; 108.2

Legend: SD: standard deviation; Min: minimum measured value; Max: maximum

measured value.

The difference between the control and the patient group was significant for the reference system and the smartphone (P< 0.01).

The difference between the reference system and the smartphone was non-significant for the control (P = 0.16) and for the patient group (P = 0.81).

### 3.3. Measurement reproducibility

**Fig 4:**
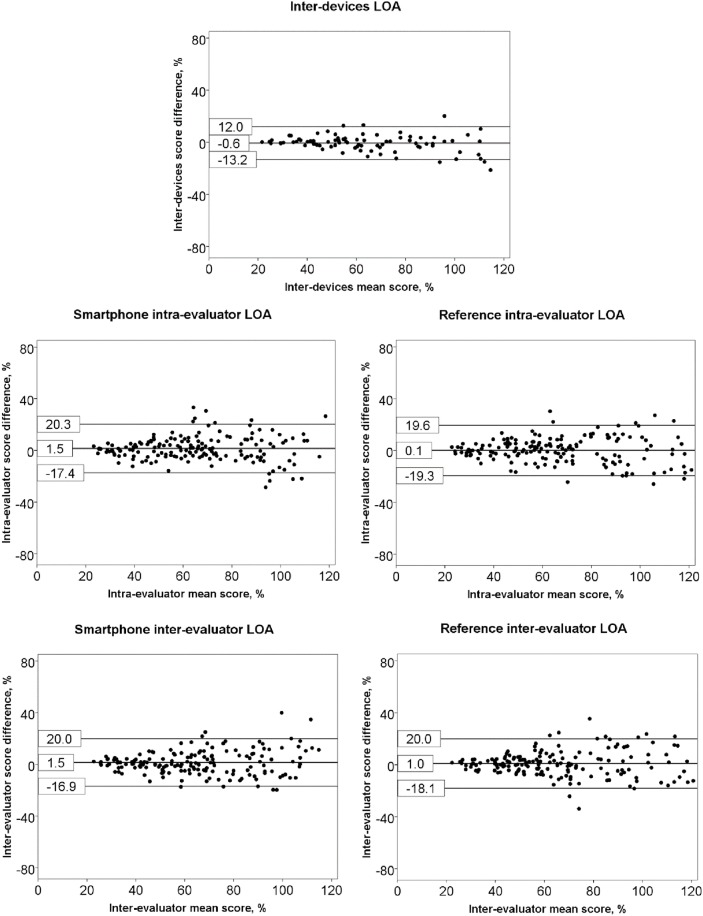
Bland and Altman graphs for inter-devices, intra- and inter-evaluator limits of agreement. Legend: LOA: limits of agreement.

The Shapiro-Wilk and Komolgorov-Smirnov tests confirmed the normal distribution of data (P > 0.05) in the patient and in the control group, regardless of device. The numerical and graphical presentations of reproducibility of measurement for inter-devices and intra- and inter-evaluator comparison are presented in Table 3 and Fig 4.

**Table 3:** Inter-devices and intra- and inter-evaluator reproducibility of the measurements.

	**ICC (95% CI)**	**LOA (%)**	**Bias (95% CI)**	**ME (%)**	**SEM (%)**
**Inter-devices**	0.97 (0.94–0.98)	-13.2 to 12.0	- 0.6 (-0.9 to 1.1)	0.7	4.0
**Intra-evaluator**					
**Smartphone**	0.92 (0.89–0.94)	-17.4 to 20.3	1.5 (0.0 to 2.9)	0.7	6.6
**Reference System**	0.92 (0.89–0.94)	-19.3 to 19.6	0.1 (- 1.4 to 1.6)	0.8	6.6
**Inter-evaluator**					
**Smartphone**	0.92 (0.90–0.94)	- 16.9 to 20.0	1.5 (0.1 to 3.0)	0.7	6.6
**Reference System**	0.93 (0.91–0.95)	- 18.1 to 20.0	1.0 (-0.5 to 2.4)	0.7	6.4

ICC: intraclass coefficient of correlation; 95%CI: 95% confidence interval; LOA: limits of agreement; ME: measurement error; SEM: standard error of measurement

### 3.4. Clinical questionnaires

The results of shoulder function, pain and quality of life questionnaires are presented in Table 4.

**Table 4:** Clinical questionnaires results.

**Questionnaires mean (SD)** *	**Patient**	**Control**
**Min;max**	(n = 65)	(n = 20)
**Constant Score (SD), points**	42.8 (17.9)	93.7 (6.6)
	**10 ; 85**	80 ; 100
**Relative Constant Score (SD), %**	55.5 (23.9)	97.6 (7.5)
	**12 ; 110**	82; 108
**SST (SD), points**	4.6 (3.1)	11.9 (0.2)
	**0; 12**	11; 12
**QuickDASH (SD), %**	42.8	1.1 (2.5)
	**0.0; 86.4**	0.0; 6.8
**VAS pain (SD), mm**	40.5 (24.2)	0.9 (2.7)
	**0; 81**	0.0; 10
**EQ-5D (SD), index**	0.70 (0.19)	1.00 (0.00)
	**- 0.18; 1.00**	1.00; 1.00
**EQ-5D VAS (SD), points**	74.3 (18.0)	98.4 (44.9)
	**10.0; 100.0**	85.0; 100.0

* Best possible scores: Constant 100 points, Relative Constant theoretically no limit (scores in % based on an age-and sex-matched normal population for Constant score), SST 12 points; QuickDASH 0, VAS pain 0, EQ5D 1.00 (index score of a value set derived from the general population sample), EQ5D VAS 100.

## 4. Discussion

This study focused on the development and validation of the shoulder function B-B Score measured by means of a smartphone. Using shoulder function scores derived from a dedicated smartphone application, the study aimed at the technical and clinical validation of them within various shoulder pathologies. Provided that the score is valid, it can offer a valuable alternative to concurrent assessment methods as it is accessible and quickly performed.

### 4.1. Devices comparison

The reference system (Physilog^®^) and the smartphone produced comparable B-B Score outcomes regarding group measurements. Although the specificities of the measurement systems were different, e.g. sensors noise, sensor ranges and sampling frequency, the smartphone performance appeared to be sufficient for the scores’ proper measurement. The mean differences between the devices were non-significant and of limited magnitude (0.0% for the patient group and 2.9% for the control group). These differences are minor in proportion to the 42.9% and 40% difference between the patient and the control group, for the reference system and the smartphone, respectively.

An excellent relationship was found between measurements from the devices (ICC 0.97). Moreover, the Bland and Altman analysis demonstrated that the systematic error of the smartphone was minor. The ME and SEM were acceptable when considered in relation to the minimum-maximum range of the scores in the study sample. Conversely, the LOA exceeded the 10% criterion that had defined the threshold. Thus, the Physilog and the iPod are interchangeable for group measurement, but the magnitude of the LOA might preclude the devices’ routine exchange.

### 4.2. Groups’ comparison

There were no deviations away from the planned sampling for this study. No significant difference was observed between the groups, except for age. The control group was purposefully younger than the patient group as it was of primary importance that the reference population had healthy shoulders. The patient characteristics were representative of the population commonly treated for shoulder pain [1, 48].

The B-B Score difference between the control and the patient groups was highly significant regardless of the device. Hence, the B-B Score clearly discriminated the patient group from the healthy group.

### 4.3. Score reproducibility

The intra- and inter-evaluator reproducibility was excellent (0.92 to 0.93) and comparable between devices. As shown by the non-significant difference between B-B Scores computed from reference and smartphone devices and by the small bias (<1.5%) derived from the Bland and Altman analyses, the B-B Score’ replication and the evaluator biases were relatively minor, indicating that the systematic errors were negligible.

Conversely, for both devices, the LOA for the repeated measurement of a B-B Score had exceeded an arbitrary 10% threshold defining its clinical utility. Thus, the results are comparable between replications and between evaluators for group measurement, but divergences are possible for single measurements when using this study’s protocol, i.e. when taking the mean of three repetitions. Measurements relating to the assessment of a single patient is still feasible but would be expected to require acquiring the mean of more than three replications in order to counteract inflated error and establish the requisite precision of measurement [49], as the variability and error in a measurement mean score decreases with the square root of the repetitions number (assuming a normal distribution of error). The simplicity of the procedure for assessing the B-B Score facilitates measurement repetition and largely overcomes this limitation.

### 4.4. Comparison with clinical scores

The kinematic measurements were also compared to currently-used clinical scores for benchmarking. The clinical scores included shoulder function (Constant, Relative Constant, SST and QuickDASH), pain (VAS) and quality of life (EQ-5D).

In healthy subjects, both clinical questionnaires and the kinematic B-B score were near to the maximum performance for all scores, showing that the reference population had almost perfect shoulder function. For patients, the observed importance of shoulder function loss was also comparable between questionnaires and the B-B score, all scores indicating a substantial function loss in the measured sample. It appeared thus in this study that the B-B score produces coherent results to the shoulder function questionnaires in terms of measured loss of function, regardless of the device used.

These results were in line with published results on the relationship between kinematic scores and clinical questionnaires, which showed moderate to high correlations of the B-B score with the Constant and SST scores and moderate correlations with the QuickDASH for various shoulder pathologies [29].

### 4.5. Body-worn sensors shoulder function evaluation in the literature

Most previous studies that had investigated the measurement properties of body-worn sensors for shoulder function scores used dedicated inertial-based system [11, 25, 26, 28, 50–55]. All these studies concluded that the inertial-based systems produced a valid evaluation of shoulder function. Similar conclusions have since been drawn by a study using smartphone technologies [29]. However, no comparison with a reference system was reported. To our knowledge the present study has been the first to investigate the concordance and the relationship of a smartphone-based and a reference inertial-based system for shoulder function evaluation. The results are valuable for research and clinics as they demonstrate that the validity of the B-B Score measurement is not altered when using a simple and accessible device.

### 4.6 Study limitations and further developments

The results apply for a situation in which the measurement has been performed under supervision and at the patient’s self-selected speed of movement. Further investigations are needed to determine the validity of the score in other conditions. For example, the relationship between devices might be different if the patients perform movements associated with the B-B Score at their maximum speed due to the difference in sensors’ characteristics. Measurement’ reliability might also be different if the patient performs the test without supervision.

The results were not detailed for each pathological subgroup in this study. This is a minor limitation with regard to the study’s objectives, as the relationship between devices is not likely to be significantly influenced by the pathology. Conversely, the use of a larger group had the advantage of providing more precise estimations of the reproducibility.

Despite the widespread use and the convenience of smartphones, there are also limitations in their use for scientific measurement. The precise features of the device are not fully disclosed by manufacturers due to commercial sensitivities. The users should remain conscious that the characteristics may differ according to smartphone version and brand. An accessible middle-segment smartphone model had been chosen specifically to offer insight into its performance' characteristics. The B-B Score would probably remain robust when faced with minor variations in smartphone technology, as it would have compared the performance of the affected shoulder with that of the healthy one [28], with the score unaffected by systematic errors in measurement affecting both sides.

Based on this study and the body of literature on the subject, it appears that smartphones most likely present measurement properties that are compatible with research requirements for measurements comparing both sides and for range of motion measurements [21–23]. Nevertheless, the validity of using smartphones for more complex measurements, e.g. those associated with 3D kinematic analysis of sport activities, remains unknown to date. Also, the aforementioned variations in smartphones’ features imply that further research is needed to investigate and quantify the influence of these variations on the outcome before clinical implementation.

The duration required to conduct the whole procedure using the smartphone was around two minutes. All things being equal, the advantage of the measurement approach used in this study mainly resides in its clinical practicality and low cost. Further development of the smartphone approach is possible to accrue maximum benefit from it clinically. Thus, an android version of the application has recently been made available to the public [56]. Future development may also consider facilitating the communication of clinically-relevant results between stakeholders, producing progression curves of functional improvements and comparing the patient's evolution of performance during care-pathways to benchmark results on a routine basis.

## 5. Conclusion

This study aimed at the technical and clinical validation of a B-B Score smartphone application for shoulder function evaluation. The results showed that the B-B Score acquired by means of a smartphone was valid and reproducible for the measurement of shoulder function of groups of patients including those presenting with rotator cuff conditions, proximal humerus fractures or adhesive capsulitis. It displayed excellent intra- and inter-evaluator reproducibility and discriminative power. Conversely, single measurements may offer reduced precision in some circumstances. The assessments acquired using either a smartphone or a reference inertial system displayed comparable measurement properties across a wide-range of clinimetrics.

Thus, the B-B Score measured with a smartphone allows valid, user-friendly and low-cost evaluation of shoulder function for research and clinical work. This could facilitate the use of objective measurement methods in routine practice and thus improve the quality of patient follow up. Further research is needed to investigate the influence of the specific characteristics of various smartphone models on results. Further technological developments are also required to achieve maximum benefit from the smartphone approach.
